# Cell-Wall-Engineered Wood Enabling Metal-and-Adhesive-Free Cross-Laminated Timber

**DOI:** 10.34133/research.1355

**Published:** 2026-07-09

**Authors:** Shang Zhang, Xuanlei Li, Xuqian Chen, Rongwen Huang, Huajie Shen, Chunwang Yang, Yuwen Zhang, Susu Yang, Jian Qiu, Yushan Yang, Lin Liu

**Affiliations:** ^1^ College of Material and Chemical Engineering, Southwest Forestry University, Kunming 650224, Yunnan, People’s Republic of China.; ^2^ Yunnan Forestry Technological College, Kunming 650224, Yunnan, People’s Republic of China.; ^3^ Machinery and Transportation College, Southwest Forestry University, Kunming 650224, Yunnan, People’s Republic of China.; ^4^ School of Design, Fujian University of Technology, Fuzhou 350118, Fujian, People’s Republic of China.

## Abstract

Lightweight structural materials that combine high strength, machinability, and sustainability are essential for next-generation construction. Wood, a renewable and low-carbon resource, offers excellent mechanical performance but remains limited by fastener stability and reliance on metals and adhesives. Here, we report a superstrong engineered wood produced through synergistic cell-wall engineering across cellular and molecular scales, enabling metal-free and adhesive-free cross-laminated timber. Pressure-controlled densification induces near-complete closure of open cells in natural beech wood, generating a dense, smooth architecture with a 3-dimensional mechanical interlocking network analogous to micro-mortise-and-tenon joints. The resulting material preserves the intrinsic anisotropic architecture of wood while forming a 3-dimensional mechanical interlocking network resembling micro-mortise-and-tenon joints. Wooden structural material exhibits substantially improved dimensional stability and mechanical performance, including a density of 1.33 g cm^−3^, a flexural strength of 150.89 MPa, a compressive strength of 88.48 MPa, and an exceptional Janka hardness of 18.84 kN. Wood-derived nails fabricated from this material function comparably to steel fasteners, providing high strength-to-weight ratios while eliminating corrosion and adhesive contamination. This approach advances timber construction toward higher performance, reduced environmental impact, and improved sustainability, offering a scalable pathway for green building applications.

## Introduction

The global construction sector is undergoing a profound transition toward low-carbon, resource-efficient, and circular material systems [1–3]. Conventional structural materials, particularly steel and concrete, remain indispensable in modern infrastructure but are associated with high embodied energy, intensive carbon emissions, and limited end-of-life circularity [4–7]. In this context, lignocellulosic materials such as wood and bamboo have attracted increasing attention as renewable, carbon-storing, and mechanically efficient alternatives for next-generation construction [8,9]. Among engineered timber products, cross-laminated timber (CLT) has emerged as a particularly promising structural platform because of its high strength-to-weight ratio, dimensional stability, prefabrication compatibility, and its ability to substantially reduce embodied carbon compared with conventional mineral- and metal-based building systems [10,11].

Despite these advantages, the full sustainable potential of CLT remains constrained by its reliance on nonbiological auxiliary components, especially petroleum-derived resins and metallic fasteners. Synthetic resins used for interlayer bonding may release volatile organic compounds, complicate recycling, and hinder the development of fully circular timber assemblies [12]. Concurrently, metal fasteners introduce material heterogeneity, thermal bridging, corrosion susceptibility, and long-term interfacial degradation, particularly under humid or chemically aggressive service environments [13,14]. These limitations are not merely secondary technical issues but constitute fundamental barriers to realizing of fully bio-based, recyclable, and durable timber structures [15]. Therefore, the development of high-performance, all-wood connection systems capable of simultaneously ensuring structural reliability, environmental compatibility, and scalable manufacturability represents a critical challenge in sustainable construction materials.

Wooden nails represent one of the earliest and most material-compatible fasteners used in timber construction and furniture fabrication [16]. Unlike metallic connectors, wooden fasteners are derived from renewable lignocellulosic resources and exhibit intrinsic hygrothermal compatibility with surrounding timber components [17,18]. At the scale of modern timber construction, such seemingly minor components collectively contribute substantial carbon footprint [19]. The lifecycle carbon emissions of a single ordinary steel nail, from iron ore mining and steel smelting to processing and forming, exceed 5 times its own weight [20]. Wooden nails reduce the introduction of heterogeneous materials into wood-based assemblies, thereby facilitating recycling, biodegradation, and closed-loop material circulation [21]. Consequently, from a sustainable construction perspective, wooden nails are not only auxiliary components but also key enabling elements for mono-material timber systems with reduced environmental impact and improved lifecycle circularity.

Nevertheless, conventional wooden nails remain limited by their intrinsic material properties, including mechanical strength, dimensional stability, and durability, which depend strongly on wood species, density, moisture content, fiber orientation, and manufacturing precision [22]. In humid or chemically aggressive environments, ordinary wooden nails may undergo swelling, biological degradation, strength loss, or interfacial loosening, thereby compromising the long-term reliability of timber connections [8,23]. These drawbacks restrict their application in modern engineered wood systems, particularly in structural contexts demanding high load-bearing capacity, stable fastening performance, and industrial-scale manufacturability.

Recent breakthroughs in wood cell-wall engineering offer a pathway to overcome these limitations by reconstructing the hierarchical structure of natural wood from the cellular to molecular scale [8,24]. Techniques such as chemical modification, resin impregnation, and pressure-controlled densification can regulate cell lumen closure, cellulose microfibril alignment, interfacial bonding, and molecular interactions among cellulose, hemicellulose, lignin, and reinforcing agents [25,26]. Such multiscale structural regulation transforms natural wood into a dense, strong, dimensionally stable, and machinable structural material, providing a compelling basis for precision-engineered wooden nails with enhanced mechanical performance and connection reliability [27,28].

Building on these advancements, the present study employs synergistic cell-wall engineering to produce ultra-strong engineered wooden structural material (WSM) specifically designed for metal-free, adhesive-free CLT manufacturing assemblies. The resulting WSM exhibits a compressive strength of 88.48 MPa and a flexural strength of 150.89 MPa, and nearly doubles the density of natural beech wood (NBW) to 1.33 g/cm^3^ while achieving exceptional Janka hardness (18.84 kN) and improved water resistance. Based on this material, precision-machined wooden nails with sharp geometry, high tip taper, and excellent holding performance are fabricated, enabling WSM nail NCLT (wNCLT) components free of adhesives and metallic fasteners while achieving a shear strength exceeding 900 N. Unlike previous studies that mainly focused on improving the intrinsic hardness, cutting ability, or impact resistance of densified/hardened wood, this study aims to translate cell-wall-engineered wood into a structural fastening system for timber construction. The novelty of this work lies in integrating pressure-controlled cell-wall engineering, precision machining of WSM wooden nails, and connection-level validation in metal-free and adhesive-free CLT assemblies. In this way, the engineered wood is not only evaluated as a high-strength material but also used as a functional connector that enables all-wood structural assembly.

## Results and Discussion

### Design principle and development of WSM

**Fig. 1.**
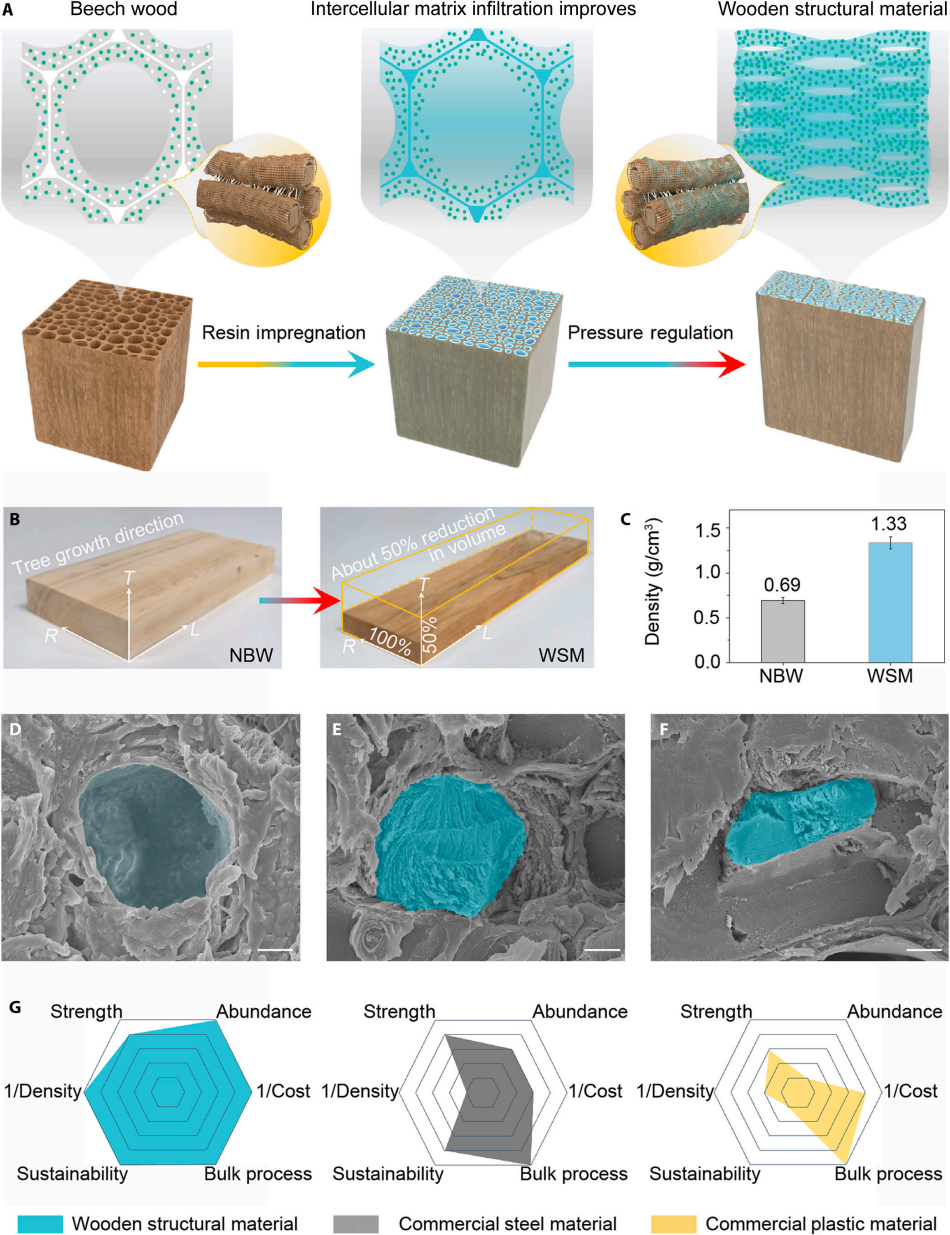
Processing approach and mechanical performance of WSM. (A) Schematic illustration of the fabrication of WSM. (B) Experimental snapshot of design principle of WSM. (C) Densities of the NBW and WSM. (D) SEM image of NBW. (E) SEM image of NBW with resin impregnation. (F) SEM image of WSM. (G) Radar plots comparing the performance of WSM, commercial steel material, and commercial plastic material

Figure 1A illustrates the processing strategy for converting raw NBW into WSM. Initially, melamine-formaldehyde resin (MUF) is injected into the cell-wall cavities of the NBW. Subsequently, the NBW sample is compressed perpendicular to the wood grain at 135 °C, which squeezes the vessels, lumens, and pits, thereby achieving further compression and densification. As shown in Fig. 1B, during this pressure-controlled densification process, uniform shrinkage occurs across the lateral areas, effectively eliminating the vast majority of pores within the NBW. This process results in a 50.0% reduction in the volume of the NBW and nearly doubles its density to 1.33 g/cm^3^, as shown in Fig. 1C. The remarkable densification of WSM is closely associated with the controlled processing parameters, including a resin loading of ≥7 wt %, an initial weight gain rate of approximately 7.08%, and a compression ratio of 55.6%, all of which contribute to the formation of a dense and mechanically interlocked structure. The densification technique increases the wall-to-lumen ratio of the NBW samples, enhances their solid density, and strengthens interactions within the cellulose framework. The result is a fully densified, ultra-strong, ultra-hard, and compact WSM characterized by an ordered arrangement of cellulose nanofibers. This enhancement is primarily attributable to the dramatically densified material structure of the NBW, which substantially promotes hydrogen bond formation between adjacent cellulose fibers within the WSM.

To investigate the microstructural evolution during NBW formation, scanning electron microscopy (SEM) was used to observe NBW before and after resin impregnation and pressure-controlled densification. As shown in Fig. 1D to F, the original porous structure of NBW was effectively filled with MUF resin during impregnation. Subsequent hot pressing caused the wood cell walls to deform, collapse, and interlock tightly, resulting in a highly compact WSM structure. In this process, MUF deposited on cell-wall surfaces may interact with cellulose fibrils through hydrogen bonding and covalent bonding, further improving interfacial integrity. Meanwhile, the chemical composition and crystallinity of NBW and WSM were further characterized by Fourier transform infrared spectroscopy (FTIR; Figs. S1 and S2), and their hierarchical architecture was analyzed in detail (Figs. S3 to S5 and Table S1). Overall, this cell-wall-engineering strategy based on resin impregnation and pressure-controlled densification preserved the inherent anisotropic structure of wood while enhancing interactions among lignocellulosic components. Consequently, WSM achieved a flexural modulus of up to 15.95 GPa (Fig. 1G), demonstrating its potential as a sustainable structural material for applications traditionally dominated by polymers, metals, and polymer composites.

### Structural characterization

**Fig. 2.**
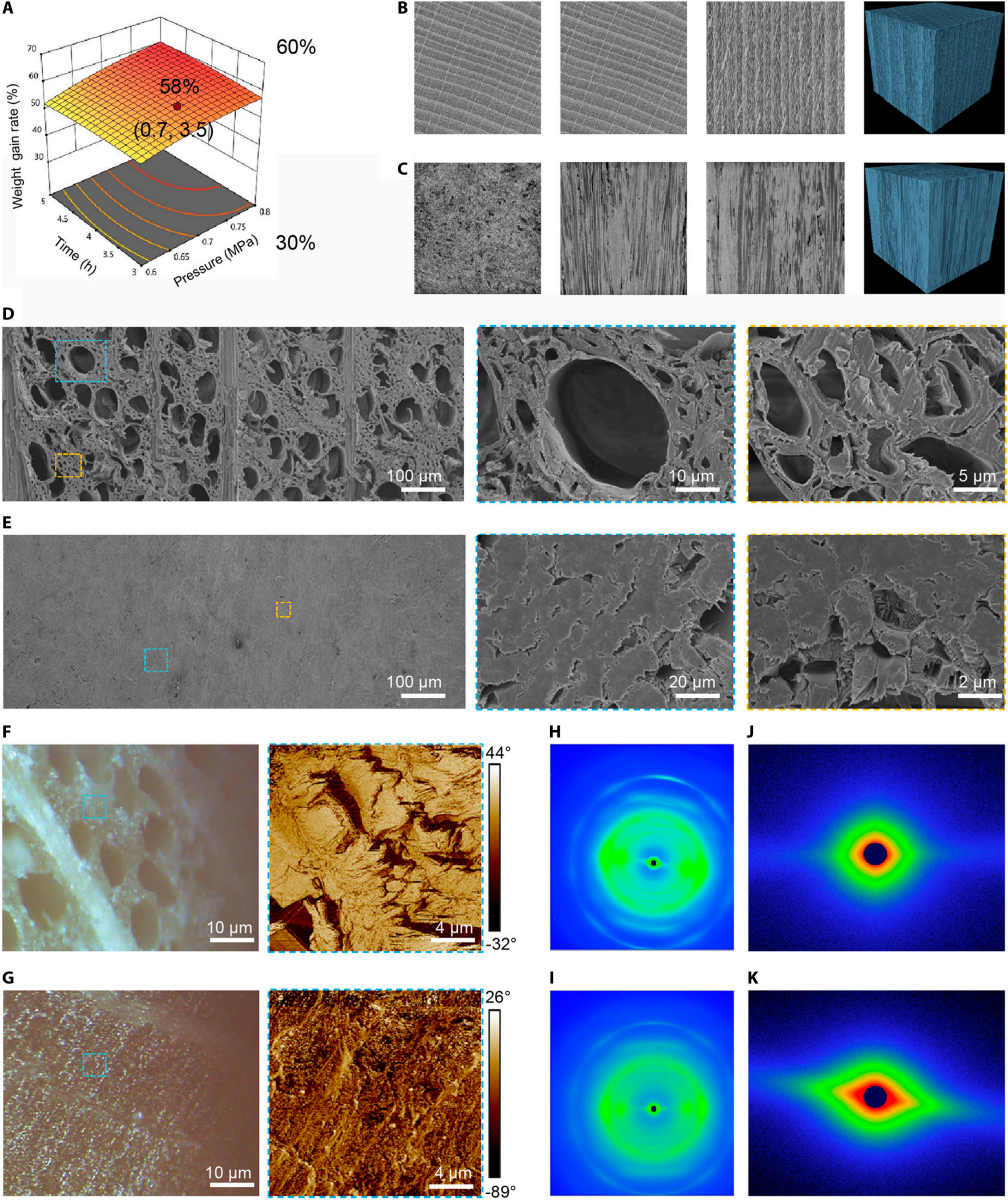
Structural characterization of WSM. (A) Response surface mapping optimization for WSM. (B) Micro-CT images of NBW. (C) Micro-CT images of WSM. (D) SEM image of NBW, which contains vessels and fibers. (E) SEM image of WSM, which contains dense wood cell wall featuring fibril density. (F) Experimental snapshot and AFM images of NBW. (G) Experimental snapshot and AFM images of WSM. (H) SAXS pattern of NBW. (I) SAXS pattern of WSM. (J) 2D WAXD patterns of NBW. (K) 2D WAXD patterns of WSM.

Conventional experimental design and optimization methods do not provide intuitive graphical representations. Orthogonal experimental design effectively addresses response issues influenced by multiple factors and levels while also indicating directions for optimization. However, because orthogonal designs rely on linear models, the optimal points cannot be intuitively observed. Although optimal values can be identified, visually discerning the optimization region or performing more detailed refinements remains challenging. Consequently, utilizing response surface methodology yields functional equations, highlighting its advantages in optimization design, as shown in Fig. 2A.

Correlation analysis between various factors and weight gain rate was conducted. The most influential factors—pressurization time and pressure—were selected for process optimization, employing a 2-factor, 5-level experimental design. Subsequently, Design-Expert software was used for model fitting and variance analysis. Weight gain rate was set as the response variable, and multiple linear regression and binomial fitting were performed for pressurization time and pressure, respectively. Results indicated that the experimental model exhibited high significance (*F* = 57.79, *P* < 0.05), with the equation fitting the experimental data well. The *F* value for the dissimilarity term was 5.26 (*P* = 0.0093), indicating insignificant dissimilarity and minimal interference from unknown factors on the experimental results. Both factors exerted significant influence (*P* < 0.05), with the order of significance for WSM preparation being pressurization time > pressurization pressure. The model’s coefficient of determination *R*^2^ = 0.8927, enabling reasonable analysis of experimental results for prepared liposomes using this model equation. Through Design-Expert software analysis based on a binomial fitting model, the optimal WSM preparation parameters were determined as weight gain rate of 58%, pressurization time of 3.5 h, and pressurization pressure of 0.7 MPa. The predicted values for weight gain rate/pressurization time and pressurization pressure were 57.59%, 3.2 h, and 0.56 MPa, respectively.

Micro-computed tomography (CT) analysis was employed to examine the pore distribution and degree of compaction of WSM, as shown in Fig. 2B and C. The results from multi-angle projection indicate that WSM exhibits a denser structure than NBW, revealing the spatial distribution of pores within the porous material. Computer-reconstructed 3-dimensional images of the material’s interior visually illustrate the location, shape, size, and distribution of voids. These images clearly reveal the microstructure of completely collapsed lignin cell walls, which are denser, more uniform, and interconnected. This finding confirms the uniform penetration of resin into the wood cell walls, creating a mechanically interlocked network structure within the cell walls that enhances interfacial bonding strength. Additionally, SEM was utilized to examine the microstructures of NBW and WSM, offering deeper insights into their process–structure–property relationships (Fig. 2D and E).

Due to the removal of MUF and the pressure-controlled process, the open cells in NBW became nearly completely sealed during compression, resulting in a highly compact structure, as shown in Fig. 2E. Subsequently, further visualization and analysis of the microstructures of NBW and WSM were performed using optical microscopy in conjunction with atomic force microscopy (AFM) images, as presented in Fig. 2F and G. The results revealed that after compression and compaction, NBW exhibited an enhanced modulus and formed a dense, smooth structural space. This enhancement is attributed to the wood treated by this process, which is composed of numerous highly curved cellulose nanofibers, with the majority of pores removed during the pressure-controlled process. Furthermore, cellulose microfibrils within the wood cell walls typically function as load-bearing units during cell compression. Subsequently, wide-angle x-ray diffraction (WAXD) was employed to evaluate the orientation index and crystallinity index of NBW and WSM samples using 2D wide-angle x-ray scattering measurements (2D-WAXS) patterns (Fig. 2H to K). The results indicate that both NBW and WSM exhibit typical cellulose Iβ crystal structures; however, WSM demonstrates a higher microfibril orientation index of 0.93. This phenomenon arises because NBW retains its structure during processing while its open pore network closes almost entirely, resulting in fibers packing more tightly together. This dense, highly ordered rearrangement enhances inter-fiber interactions at the micro-level through increased crystallinity, thereby strengthening the mechanical properties. Concurrently, small-angle x-ray scattering (SAXS) comparisons of NBW and WSM revealed that WSM exhibited a more pronounced equatorial stripe scattering pattern, indicating that the oriented arrangement of cellulose fibers facilitates efficient load-bearing.

### Physical and mechanical property characterization

**Fig. 3.**
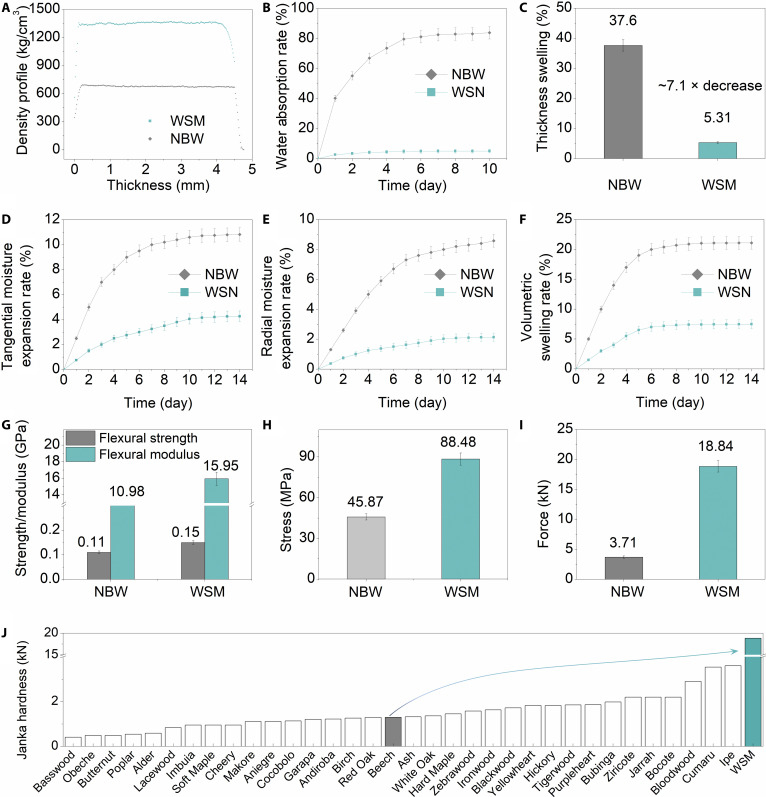
Physical and mechanical property characterization of WSM. (A) Density profile and its distribution of NBM and WSM. (B) Water absorption rate curves of NBM and WSM. (C) Thickness swelling of NBM and WSM. (D) Tangential moisture expansion rate curves of NBM and WSM. (E) Radial moisture expansion rate curves of NBM and WSM. (F) Volumetric swelling rate curves of NBM and WSM. (G) Flexural strength and modulus of NBM and WSM. (H) Axial compressive strength and modulus of NBM and WSM. (I) Janka hardness of NBM and WSM. (J) Exotic and domestic lumber Janka values.

As shown in Fig. 3A, the peak values of the cross-sectional density curves for NBW and WSM correspond to their respective density values. Nevertheless, the dense structure of WSM and the systemic effect of MUF resin permeating the cell walls confer exceptional dimensional stability. This is attributed to the extensive pore structure and hydrophilic fibers within NBW, which can absorb large amounts of water. In contrast, following the addition of MUF and the densification treatment, WSM inhibits water penetration within the wood, resulting in extremely low moisture content (Fig. 3B). The results demonstrate that WSM exhibits a superior water absorption and thickness swelling compared to NBW, with an average reduction of 93.98%. Additionally, MUF resin forms a 3-dimensional mechanical interlocking network that resembles “micro-mortise-and-tenon joints” between cell gaps, within cells, and inside cell walls. Following the pressure-controlled process, it compacts most vessels and wood cells, demonstrating interactions across various scales. This characteristic confers water repellency, achieving exceptionally low water absorption thickness expansion (Fig. 3C) and extremely low radial, tangential, and volumetric swelling rates (Fig. 3D to F). Furthermore, the static flexural strength, tensile strength, surface hardness, and other properties of NBW and WSM specimens were characterized and analyzed. The results indicate that the maximum load-bearing capacities of NBW and WSM specimens were approximately 0.11 ± 0.01 GPa and 0.15 ± 0.02 GPa, respectively (Fig. 3G and Fig. S6), with WSM exhibiting the highest flexural strength. Figure 3H and Fig. S7 display the elastic moduli of NBW and WSM. The results indicate that WSM exhibits a flexural modulus of 15.95 ± 0.75 GPa, which was significantly higher than that of NBW (*P* < 0.05).

The enhanced mechanical performance of WSM can be attributed to the synergistic effects of resin impregnation and compression densification, where controlled resin loading, weight gain rate, and compression ratio collectively promote the formation of a highly compact and interconnected microstructure. This enhancement is attributed to the MUF resin, which effectively removes most pores through pressure-controlled removal in intercellular spaces, within cells, and within cell-wall cavities, thereby forming a 3-dimensional mechanical interlocking network structure that resembles “micro-mortise-and-tenon joints”. Moreover, characterization calculations reveal that WSM exhibits a 93.68% increase in parallel-grain compressive strength compared to NBW. The dense structure of this material provides resistance to penetration by rigid bodies. The resistance encountered when a hemispherical steel indenter of specified radius is pressed into wood under static load serves as an indicator of its hardness. In this study, a hemispherical steel indenter with a radius of 5.64 ± 0.01 mm was uniformly pressed into the center of the test surfaces of both NBW and WSM specimens until a penetration depth of approximately 5.64 mm was achieved (Fig. S8). This is attributed to the MUF resin, which removes most pores through pressure-controlled removal in intercellular spaces, within cells, and within cell-wall cavities, forming a 3-dimensional mechanical interlocking network structure resembling “micro-mortise and tenon joints”. The results indicate that WSM has a Janka hardness value of 18.84 ± 0.94 kN, which is 5.1 times greater than that of NBW (Fig. 3I). As shown in Fig. 3J, Janka hardness of WSM not only surpasses exotic and domestic lumber, including Fagis, Quercus Russian, white oak, hickory, Canarywood, Bloodwood, red sandalwood, and Snakewood [27,28], but also exceeds that of metallic materials such as aluminum alloy, stainless steel, and carbon steel, as well as engineering materials including carbon fiber reinforced polymer, glass fiber reinforced plastic, polymethyl methacrylate, and wood–plastic composite [29–34]. Its hardness is comparable to that of other high-quality densified woods [8,24–26,35].

### Engineering application of lightweight wooden nail cross-laminated timber

Wooden structures are globally favored due to their superior thermal properties, excellent ecological adaptability, and low carbon emissions. CLT is a new type of engineered wood material created by cross-bonding multiple layers of wood panels with alternating grain orientations. This process substantially enhances the dimensional stability and mechanical properties of the wood, making CLT widely applicable in mid-to-high-rise timber buildings. However, traditional CLT production relies on metal fasteners, such as nails and bolts, and synthetic adhesives for interlayer connections and fixation, which often contain volatile organic compounds like formaldehyde. This not only renders the structure susceptible to corrosion and reduces its durability but also poses health risks to humans and contributes to environmental pollution. Furthermore, the cellular structure of natural wood has inherent limitations, such as restricted mechanical strength in the cell walls. This makes the material prone to fracture or deformation under heavy loads, thereby limiting the application of CLT in large-span, high-load building structures.

**Fig. 4.**
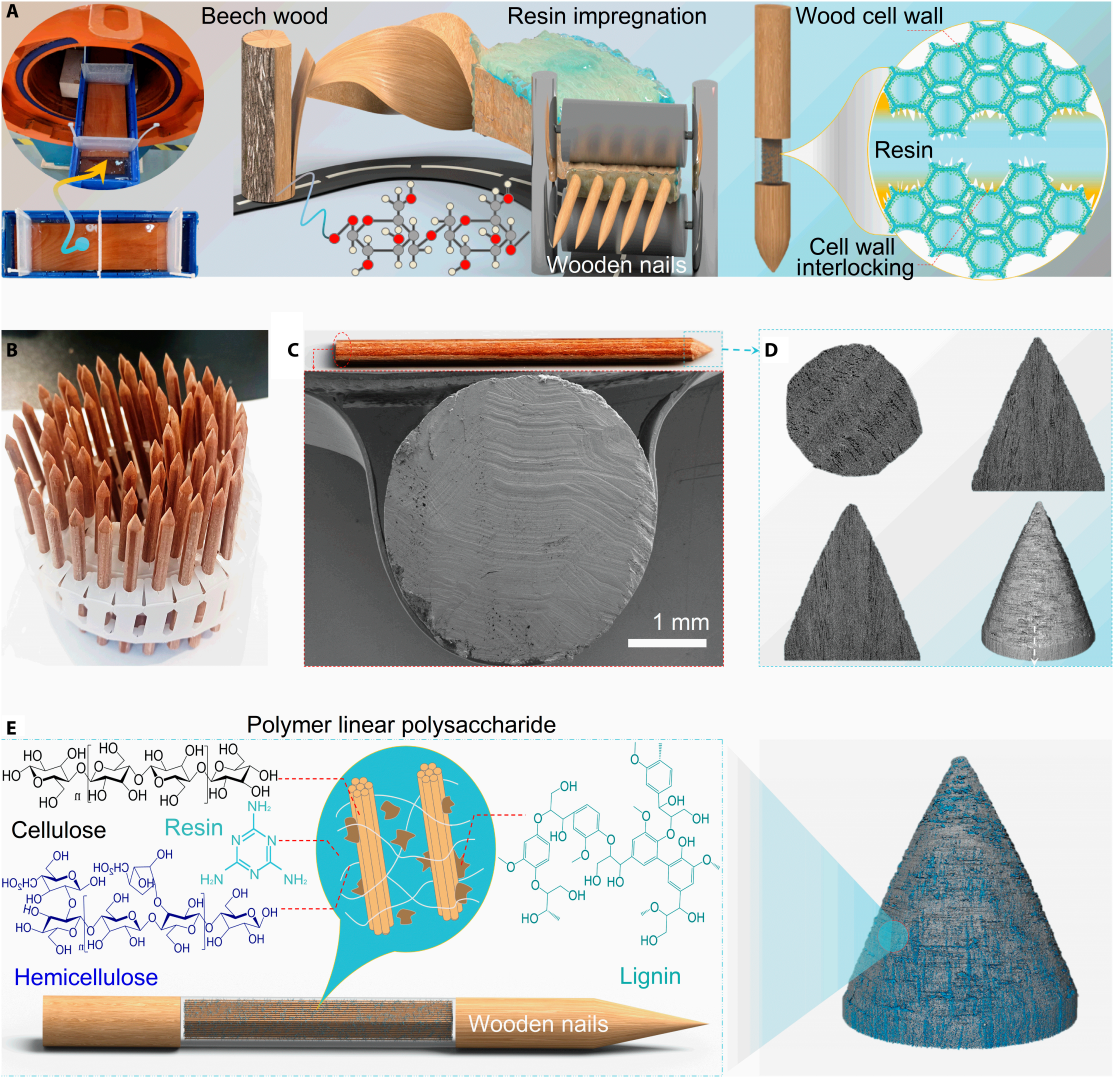
Demonstration of engineering application of WSM as lightweight wooden nail cross-laminated timber to replace adhesive or steel nails. (A) Schematic illustration of the fabrication of lightweight wooden nails and resin formed mechanical interlocks between veneer layers, inside cells, and inside cell walls. (B) Experimental snapshot of lightweight wooden nails fabricated from WSM developed in this study. (C) SEM image of lightweight wooden nail. (D) Micro-CT images of lightweight wooden nail. (E) Relationship between the composition of cellulose, hemicellulose, lignin, and resin in wood nails and their structure diagrams.

As the construction industry demands increasingly advanced material properties—particularly driven by ecological architecture and sustainable design—the integration of wooden nails with CLT walls, and the exploration of how these fasteners can incorporate multifunctionality and sustainability into modern ecological timber-framed structures, carries considerable responsibility. This approach not only ensures structural stability and safety but also highlights the advantages of wood as a renewable resource, effectively meeting diverse environmental and application requirements. By adopting this alternative approach, we can enhance the performance of wood dowels while reducing reliance on traditional timber resources, thereby advancing sustainability goals. However, conventional wood dowels fail to meet engineering demands in humid or corrosive environments—prone to decay and insufficient strength in damp conditions—directly compromising the durability and safety of timber structures [13,22,25]. Therefore, we engineered the cell walls to form mechanical interlocking at different scales, designing novel wood nails from WSM that feature 100% natural material, precise construction, and outstanding performance (Fig. 4A). As shown in Fig. 4B, the prepared WSM is polished to manufacture the wood nail, with its tip sharpened to match the precision of steel nails. Results from bending wall thickness tests indicate that WSM wood nails possess exceptionally high mechanical strength, as shown in Fig. S9.

To verify the internal microstructure and defect status of the processed wood nail, we characterized its cross-sectional microstructure using micro-CT imaging (Fig. 4C). The results reveal that the wood nail retains the densified structural characteristics of the original WSM material. As shown in Fig. 4D, further validation of the precision manufacturing technology underlying this innovation was achieved by subjecting each wooden nail to CT scanning to detect internal defects. A computerized numerical control lathe machined precise conical structures with a tip taper of 1:25, ensuring uniform stress distribution during insertion. This is due to resin-impregnated NBW undergoing pressure-controlled densification, which causes MUF deposited in cell-wall cavities to tightly bond with cellulose fibrils through covalent and hydrogen bonds. This process increases the wall-to-lumen ratio in NBW samples, enhances solid density, and strengthens interactions within the cellulose framework. This yields a fully densified, ultra-strong, ultra-hard, and compact WSM characterized by ordered cellulose nanofiber alignment, which remarkably enhances hydrogen bond formation between adjacent cellulose fibers in the wood nail (Fig. 4E).

### Characterization of wooden nail

**Fig. 5.**
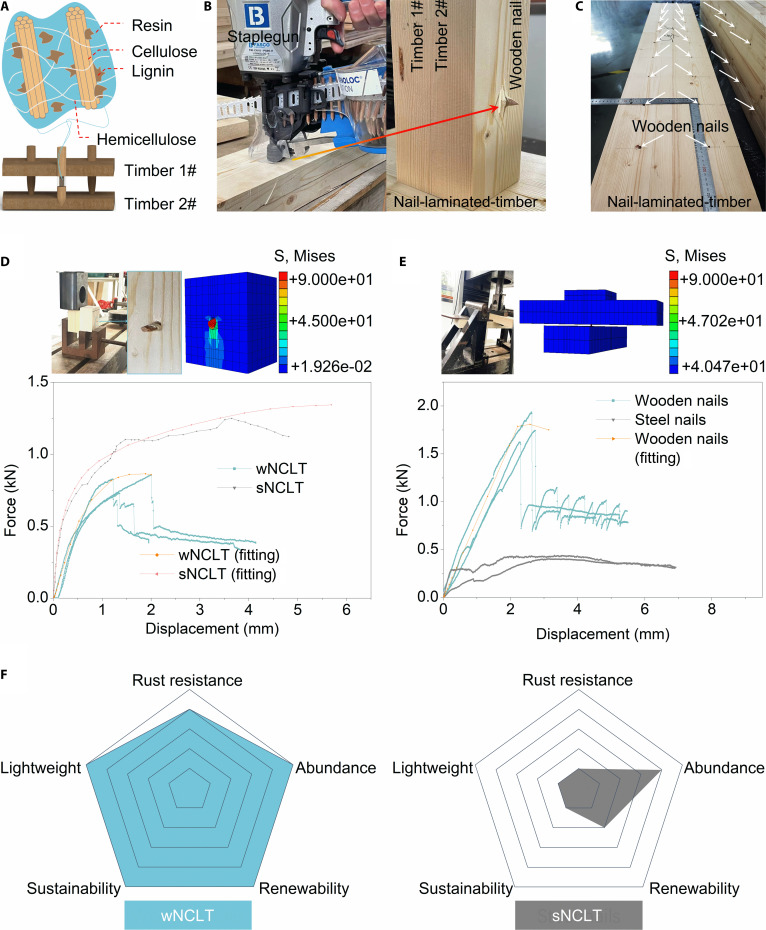
Characterization of wooden nail withdrawal test. (A) Structure diagrams of wood nails. (B) Experimental snapshot of the fabrication metal-and-adhesive-free cross-laminated timber. (C) Experimental snapshot of wNCLT. (D) Shear strength test and their corresponding finite element simulation results of wNCLT. (E) Withdrawal test and their corresponding finite element simulation results of wNCLT. (F) Radar plots comparing the advantages of wNCLT and commercial sNCLT.

The use of wooden dowels in construction enhances carbon storage. When buildings are eventually demolished, these dowels return to nature, thereby completing the closed loop of the carbon cycle. This “cradle-to-cradle” material lifecycle exemplifies the ideal model of a circular economy. Our computer numerical control (CNC)-machined WSM wood nails demonstrate that these fasteners can effectively connect timber structural components when integrated with CLT walls. To validate the efficacy of WSM nails, we utilized a smart nail to secure 2 pieces of natural timber together, thereby forming nail cross-laminated timber (NCLT), as shown in Fig. 5A and B. Figure 5B illustrates that the natural timber remains undamaged during the penetration process of the WSM dowels. Consequently, we employ this embedded woodworking strategy as a production method for large-scale CLT materials, fundamentally transforming the traditional wood construction cycle, which is often characterized by lengthy timelines and on-site disorganization. As shown in Fig. 5C, custom-produced WSM wood-nailed components function as connectors, allowing for millimeter-precise machining to produce large-scale wNCLT. This simplicity not only lowers construction costs but also facilitates future equipment maintenance. This represents a sustainable pathway that is unattainable with metal fasteners, thereby enabling the circular flow of building materials. Further shear testing was conducted on stacked natural spruce blocks secured with WSM wood nails and steel nails, utilizing wNCLT and steel nail cross-laminated timber (sNCLT), respectively. The results indicated that sNCLT achieved a maximum shear load of up to 1200 N. This high shear strength resulted from the steel nail exhibiting both buckling deformation and slip-pullout behavior during shear loading, as shown in Fig. S10. In contrast, wNCLT demonstrated minimal pull-out or buckling during shear testing, revealing a degree of brittleness in the transverse shear behavior of the wood nails (Fig. S11) while still achieving a shear strength exceeding 900 N. This phenomenon occurs because transverse shear is infrequently encountered in practical applications [16].

As shown in Fig. 5D, finite element analysis further validates its practical application value. Additionally, pull-out resistance tests combined with finite element simulation analyzed the holding force of 2 fastener materials—the resistance encountered when wooden and steel nails are pulled out of wNCLT and sNCLT, respectively. As shown in Fig. 5E and Fig. S12, the P–Δ curve during the steel nail extraction process from sNCLT exhibits a distinct linear region from the initial stage. Following the onset of peak load (0.29 kN), a gradually flattening region appears, attributed to slippage occurring during nail extraction. In contrast, the P–Δ curve for the WSM wood nail during extraction from wNCLT still exhibits a distinct linear region in the initial stage. Following the onset of peak load (1.93 kN), it shows a sharp linear decrease, followed by a pronounced linear increase to a certain peak load (1.06 kN), then a sharp linear decrease again. This pattern repeats cyclically multiple times. The increased area under the load–displacement curve indicates higher energy dissipation during nail withdrawal, which is consistent with the proposed interlocking mechanism. The enhanced withdrawal resistance is likely associated with the formation of micro-mortise-and-tenon interlocking structures at the nail–wood interface, which increase mechanical anchorage and resistance to pull-out. The results indicate that WSM wood nails exhibit superior holding power compared to steel nails. This is attributed to the instantaneous heat generated by surface friction (reaching 60 to 70 °C) during high-speed driving, which activates the thermoplastic properties of lignin, transforming it from a solid to a semi-fluid state [13,22]. This “lignin thermoplastic effect” acts like natural glue, forming a micro-mortise-and-tenon-like connection between the smooth WSM nail body and substrate and the wood (Fig. S12). The thermogravimetric analysis (TG) (Fig. S13), derivative thermogravimety (DTG) (Fig. S14), and differential scanning calorimetry (DSC) (Fig. S15) results indicate that the engineered wood retains thermally responsive lignin structures. Although transient lignin softening during nail insertion was not directly measured in the present study, the thermoplastic behavior of lignin may facilitate local interfacial adaptation under frictional heating and mechanical compression. Therefore, lignin thermoplasticity is considered a potential contributing factor, whereas the enhanced withdrawal performance is more directly supported by the observed micro-mortise-and-tenon interlocking structures, increased surface roughness, and higher withdrawal energy. The dynamic mechanical analysis (DMA) results reveal a temperature-dependent decrease in storage modulus and an increase in damping factor, indicating enhanced viscoelastic mobility of the lignin-rich amorphous phase, as shown in (Fig. S16). During pneumatic insertion, transient frictional heating and local compression may activate this thermoplastic response, allowing the surrounding wood matrix to deform and conform to the rough WSM nail surface. Upon cooling, the lignin-rich matrix can re-solidify and contribute to mechanical anchorage, thereby reinforcing the micro-mortise-and-tenon interlocking structure and increasing the energy required for nail withdrawal. Upon cooling, this creates a stable connection far exceeding mechanical interlocking. This occurs because the cell walls on the nail surface rupture under pressure, releasing lignin that permeates the intercellular spaces of the substrate wood. This forms a 3-dimensional network structure resembling “micro-mortise-and-tenon joints”.

Over time, the natural aging of lignin actually enhances the bond strength—a stark contrast to the corrosion fatigue experienced by metal fasteners. Therefore, by comparing WSM wood nails with commercial steel nails in terms of rust resistance, versatility, lightweight properties, renewability, and sustainability (Fig. 5F), WSM wood nails demonstrate considerable advantages and hold promise as a replacement for commercial steel nails. Although softwood and hardwood species possess different native anatomical structures and fiber characteristics, the densification treatment remarkable reduced the influence of these initial differences by increasing cell-wall compactness and decreasing porosity. Consequently, the densified wooden nails prepared from different species exhibited comparable flexural performance and maintained sufficient strength for connection applications. Considering that the connection response is primarily governed by the mechanical capacity of the densified nails, only wooden nails fabricated from Douglas fir were selected for the subsequent connection tests. This species showed representative mechanical performance and stable processing quality, making it suitable for evaluating the structural behavior of the proposed connection system. For clarity, beech wood, poplar wood, and Douglas fir were all included in the comparative evaluation of wooden nail performance shown in Figs. S9 to S11. To evaluate the mechanical performance of the wooden nails, comparative bending tests were conducted on nails fabricated from natural beech wood, MUF-treated poplar wood, MUF-treated Douglas fir, alkali-treated beech wood, and MUF-treated beech wood. For clarity, beech wood, poplar wood, and Douglas fir were all included in the comparative evaluation of wooden nail performance shown in Figs. S17 and S18. To evaluate the mechanical performance of the wooden nails, comparative bending tests were conducted on nails fabricated from natural beech wood, MUF-treated poplar wood, MUF-treated Douglas fir, alkali-treated beech wood, and MUF-treated beech wood.

## Conclusion

In summary, we achieved mechanical interlocking of the resin across different scales within natural wood through cell-wall engineering. Subsequently, pressure-controlled densification enabled the MUF deposited in cell-wall cavities to bond tightly with cellulose fibrils through covalent and hydrogen bonds. This enhanced the wall-to-cavity ratio in NBW samples, resulting in a fully densified, ultra-strong, ultra-hard, and densely packed WSM with an ordered arrangement of cellulose nanofibers. The resulting WSM exhibits ultra-high hardness, strength, durability, and safety surpassing untreated wood—even rivaling certain metals—offering - remarkable performance advantages for large-scale production of naturally abundant, low-cost, robust, and versatile engineered materials. Furthermore, based on the design of WSM engineering materials, a novel wood nail has been developed that is as sharp as steel nails and features an extremely high tip taper. When driven into wood at high speed, the instantaneous heat generated by surface friction activates the “lignin thermoplastic effect”. Upon cooling, this forms a molecular-level, 3-dimensional network structure that resembles “micro-mortise-and-tenon” joints between the nail and the substrate. This establishes a much more stable connection than mechanical interlocking, contrasting sharply with the corrosion fatigue associated with metal fasteners. As a metal-free, adhesive-free CLT component utilizing this “micro-mortise-and-tenon” connection, the wooden nails not only join large load-bearing elements in sustainable construction but also represent a key breakthrough toward the low-carbon development goal of metal-free timber structures. They exemplify the continuation of carbon storage in building applications and are essential for completing the carbon cycle upon building demolition. This approach eliminates safety hazards and maintenance costs associated with corrosion in timber structures over time. This technique preserves the warm, organic texture of wood while imparting concrete-like structural strength through scientific engineering. This represents an ideal circular economy model, elevating ancient woodworking techniques to become integral to modern high-tech manufacturing systems.

## Materials and Methods

### Materials

The wood species used in this study included *Fagus sylvatica* (beech wood, typical sample dimensions: 9 × 100 × 400 cm^3^), 30 years old, sourced from Romania; *Populus ussuriensis* Kom. (poplar wood, typical sample dimensions: 9 × 100 × 400 cm^3^), 16 years old, sourced from Jiangsu Province, China; and *Pseudotsuga menziesii* (Douglas fir, typical sample dimensions: 9 × 100 × 400 cm^3^), 20 years old, sourced from Alberta. The tree’s diameter at breast height of beech, poplar, and Douglas fir were 50 cm, 55 cm, and 30 cm, respectively. The air-dry densities of beech, poplar, and Douglas fir were 0.69, 0.48, and 0.53± g cm^−3^, respectively. The MUF resin, developed by Husen Trading (Yunnan) Co. Ltd., possessed the following basic parameters: a solid content of 55% to 65%, viscosity of 18 MPa·s (23 °C), and pH of 7.5 to 8.5.

### Fabrication of WSM

Firstly, wood specimens of suitable dimensions were selected and impregnated with MUF resin solution. The core objective was to ensure complete resin filling of internal wood pores until specimens sank to the solution bottom under their own weight. Under atmospheric pressure, this process typically requires over 48 h. To enhance resin penetration efficiency and uniformity, an alternating vacuum-pressure impregnation technique is employed: After placing the specimens in the impregnation reactor, the MUF solution is directly injected and pressurized at 0.6 MPa for 90 min to promote resin penetration into the wood cell cavities and cell walls; at this stage, the weight gain rate is approximately 7.08%. Excess impregnation liquid is then drained, followed by 30 min of vacuum treatment at 0.06 MPa to remove unbound free resin, further optimizing resin distribution uniformity within the wood. To achieve the desired MUF resin content (≥7%), the wood samples were removed and leaked for 4 min to remove excess MUF resin, and the resin content of the wood samples prepregs was calculated according to the following equation: *G*_2_ = *G*_1_ + (*G*_1_ (1 − *w*) × *m*)/*P* × 100%, where *G*_1_ and *G*_2_ are the weights of the wood samples before and after immersion in the MUF resin, respectively; *m* is the MUF resin content; *P* is the solid content of the MUF resin; and *w* is the moisture content of the wood samples before immersion. The as-prepared above resin-impregnated wood samples are transferred to a mechanical hot press for densification. The slab was compressed at 135 °C at 10 MPa for approximately 35 min to obtain WSM. Following this process, the wood thickness was reduced by 55.6% from its initial state (approximately 5 mm), forming resin-reinforced dense wood (WSM).

### Fabrication of WSM nails and wNCLT

WSM nails were fabricated from the prepared wood structural material using a CNC round-bar processing machine (MB9010T, Haoyu Chengda Woodworking Machinery Co. Ltd., China). The WSM was machined into cylindrical wooden nails with a diameter of 5 mm. The fabricated nails were subsequently assembled into collated nail strips using the plastic strip system of a Beck Group F44 pneumatic nail gun. For NCLT assembly, the magazine scale of the pneumatic nail gun was adjusted according to the actual nail length. Each WSM nail was smoothly inserted into the guide groove of the nail gun, with the nail head kept flush with the upper surface of the firing channel. This arrangement ensured stable positioning and consistent driving behavior during nailing, thereby improving the reliability and repeatability of the assembly and testing process.

### Characterizations

Microstructural analysis of NBW and WSM was performed via SEM (SU8010, Japan). AFM images of NBW and WSM were recorded using a Bruker Dimension Icon. All mechanical properties of NBW and WSM were determined using an electromechanical universal testing machine CMT 6104, operated at a rate of 5 mm min^−1^. Janka hardness of NBW and WSM is characterized by the load required to press an 11.28-mm steel ball into wood until it reaches a depth equal to half the ball’s height. All experiments were conducted using at least 3 independent specimens unless otherwise specified. The density of NBW and WSM was calculated by high-precision solid–liquid powder densitometer. Density measurements were performed using 10 specimens for each experimental group. The density distribution across the cross-section of NBW and WSM materials was measured using the DAX 6000 x-ray profile densitometer (GreCon, DAX6000, Germany) by scanning x-rays along the thickness direction. SAXS of NBW and WSM was used to characterize the molecular arrangement of cellulose microfibrils. 2D wide-angle X ray diffraction (2D-WAXD) of NBW and WSM was used to characterize the crystallinity and orientation index.

## Data Availability

Data will be made available on request.

## References

[1] Ding Y, Pang Z, Lan K, Yao Y, Panzarasa G, Xu L, Lo Ricco M, Rammer DR, Zhu J, Hu M, et al. Emerging engineered wood for building applications. Chem Rev. 2022;123(5):1843–1888. 36260771 10.1021/acs.chemrev.2c00450

[2] Harris NL, Gibbs DA, Baccini A, Birdsey RA, De Bruin S, Farina M, Fatoyinbo L, Hansen MC, Herold M, Houghton RA, et al. Global maps of twenty-first century forest carbon fluxes. Nat Clim Chang. 2021;11(3):234–240.

[3] Shi Q, Zhu J, Liu Z, Guo H, Gao S, Liu M, Liu Z, Liu X. The last puzzle of global building footprints—Mapping 280 million buildings in East Asia based on VHR images. J Remote Sens. 2024;4:0138.

[4] Lin W, Xing J, Zhou Y, Pan L, Yang L, Zhang Y, Liu XX, Xiong C, Li W, Sun Z. A biomimetic cement-based solid-state electrolyte with both high strength and ionic conductivity for self-energy-storage buildings. Research. 2024;7:0379. 38779490 10.34133/research.0379 PMC11109515

[5] Li C, Pradhan P, Chen G, Kropp JP, Schellnhuber HJ. Carbon footprint of the construction sector is projected to double by 2050 globally. Commun Earth Environ. 2025;6(1):831. 41163641 10.1038/s43247-025-02840-x PMC12559003

[6] Liu M, Li D, Wang X, Chen H, Zhang D. Carbon utilization in construction materials: Technologies, challenges, and opportunities. Innov Geosci. 2025;3(3):100149.

[7] Zhao Y, Ma J, Li Y, Cheng K, Zhang M, Liu Z, Yang F. Carbon emission based predictions of anthropogenic impacts on groundwater storage at typical basins in 2050. Research. 2025;8:0680. 40458612 10.34133/research.0680 PMC12129122

[8] Song J, Chen C, Zhu S, Zhu M, Dai J, Ray U, Li Y, Kuang Y, Li Y, Quispe N, et al. Processing bulk natural wood into a high-performance structural material. Nature. 2018;554(7691):224–228. 29420466 10.1038/nature25476

[9] Su J, Yang Y, Wan C, Li X, Chai Y, Chai H, Yuan J, Wu Y. A novel flame-retardant, smoke-suppressing, and superhydrophobic transparent bamboo. Research. 2024;7:0317. 38357698 10.34133/research.0317 PMC10865110

[10] Lan K, Favero A, Yao Y, Mendelsohn RO, Wang HS-H. Global land and carbon consequences of mass timber products. Nat Commun. 2025;16(1):4864. 40419537 10.1038/s41467-025-60245-y PMC12106697

[11] Vasuks P, Vamza I, Valtere M, Bezrucko T, Blumberga D. Recycled cross-laminated timber as a low environmental impact alternative to virgin material: Latvia case study. Case Stud Constr Mater. 2025;22: Article e04094.

[12] Li H, Wang L, Wei Y, Semple KE, Dai C. Out-of-plane bending behavior of cross-laminated timber members enhanced with fiber-reinforced polymers. J Build Eng. 2023;66: Article 105862.

[13] Wang S, Wang F, Kong F, Ma P, Chen Z, Que Z. Influence of repeated wetting and drying on withdrawal capacity of wooden nails and metal nails. Constr Build Mater. 2023;409: Article 133991.

[14] Zelinka SL, Rammer DR. Modeling the effect of nail corrosion on the lateral strength of joints. For Prod J. 2012;62(3):160–166.

[15] Chen C, Kuang Y, Zhu S, Burgert I, Keplinger T, Gong A, Li T, Berglund L, Eichhorn SJ, Hu L. Structure–property–function relationships of natural and engineered wood. Nat Rev Mater. 2020;5(9):642–666.

[16] Riggio M, Sandak J, Sandak A. Densified wooden nails for new timber assemblies and restoration works: A pilot research. Constr Build Mater. 2015;102:1084–1092.

[17] Ma H, Li S, Wang S, Yang W, Han J. Biomimetic all-wood sponge for the co-generation of adsorption-based atmospheric water harvesting and hydrovoltaic power generation. Research. 2026;9:1195. 41884331 10.34133/research.1195 PMC13009533

[18] Li SC, Hu BC, Ding YW, Liang HW, Li C, Yu ZY, Wu ZY, Chen WS, Yu SH. Wood-derived ultrathin carbon nanofiber aerogels. Angew Chem Int Ed. 2018;57(24):7085–7090. 10.1002/anie.201802753 29687551

[19] Meyer F, Elliot T, Craig S, Goldstein BP. The carbon footprint of future engineered wood construction in Montreal. Environ Res Infrastruct Sustain. 2024;4(1): Article 015012.

[20] Jiang J, Wang H, Lin J, Wang F, Liu Z, Wang L, Li Z, Li Y, Li Y, Lu Z. Nature-inspired hierarchical building materials with low CO_2_ emission and superior performance. Nat Commun. 2025;16(1):3018. 40148326 10.1038/s41467-025-58339-8 PMC11950366

[21] Song X, Du S, Deng C, Shen P, Xie M, Zhao C, Chen C, Liu X. Carbon emissions in China’s steel industry from a life cycle perspective: Carbon footprint insights. J Environ Sci. 2025;148:650–664. 10.1016/j.jes.2023.04.027 39095197

[22] Korte H, Koch G, Krause KC, Koddenberg T, Siemers S. Wood nails to fix softwoods: Characterization of structural deformation and lignin modification. Eur J Wood Wood Prod. 2018;76(3):979–988.

[23] Dong Y, Chen X, Lu D, Zhao X, Zhang Z, Cao J. Decoupling density–strength–toughness in wood modification via molecular compaction. Adv Mater. 2026;38(12): Article e21176. 41532189 10.1002/adma.202521176

[24] Lu Z, Qi L, Chen J, Lu C, Huang J, Chen L, Wu Y, Feng J, Lin J, Liu Z, et al. A superstrong, decarbonizing structural material enabled by microbe-assisted cell wall engineering via a biomechanochemical process. Sci Adv. 2025;11(30): Article eady0183. 40700509 10.1126/sciadv.ady0183 PMC12285728

[25] Chen B, Leiste UH, Fourney WL, Liu Y, Chen Q, Li T. Hardened wood as a renewable alternative to steel and plastic. Matter. 2021;4(12):3941–3952.

[26] Huang Y, Jiang K, He Y, Hu J, Dyer K, Chen S, Akinlabi E, Zhang D, Zhang X, Yu Y, et al. A natural lignification inspired super-hard wood-based composites with extreme resilience. Adv Mater. 2025;37(19):2502266. 40143781 10.1002/adma.202502266 PMC12075913

[27] Ross RJ. Wood handbook: Wood as an engineering material. USDA Forest Service, Forest Products Laboratory; General Technical Report FPL-GTR-190. 1999.

[28] Green DW, Winandy JE, Kretschmann DE. Mechanical properties of wood. In: *Wood handbook: Wood as an engineering material*. Madison (WI): USDA Forest Service, Forest Products Laboratory; General Technical Report FPL-GTR-113; 1999. p. 4.1–4.45.

[29] Dharani Kumar S, Magarajan U, Kumar SS, Prabhu L. Mechanical and ballistic studies of boron carbide filler reinforced glass fiber composites. Polym Compos. 2024;45(16):14953–14965.

[30] Ghasali E, Alizadeh M, Pakseresht AH, Ebadzadeh T. Preparation of silicon carbide/carbon fiber composites through high-temperature spark plasma sintering. J Asian Ceram Soc. 2017;5(4):472–478.

[31] El-Wazery M, El-Elamy M, Zoalfakar S. Mechanical properties of glass fiber reinforced polyester composites. Int J Appl Sci Eng. 2017;14(3):121–131.

[32] Yu Q, Wang Y, Ye H, Sheng Y, Shi Y, Zhang M, Fan W, Yang R, Xia C, Ge S. Preparation and properties of wood plastic composites with desirable features using poplar and five recyclable plastic wastes. Appl Sci. 2021;11(15):6838.

[33] Kaymakci A, Ayrilmis N. Investigation of correlation between Brinell hardness and tensile strength of wood plastic composites. Compos B Eng. 2014;58:582–585.

[34] Chen Y, Fu J, Dang B, Sun Q, Li H, Zhai T. Artificial wooden nacre: A high specific strength engineering material. ACS Nano. 2020;14(2):2036–2043. 31934744 10.1021/acsnano.9b08647

[35] Martins IZ, Deldotti LR, Soriano J, Faria DL. Janka hardness of hardwood species evaluated by the nondestructive sclerometric method. Mater Struct. 2022;55(9):227.

