# Supplementary material for: Cell-Wall-Engineered Wood Enabling Metal-and-Adhesive-Free Cross-Laminated Timber
Source: Research (Wash D C). 2026 Jul 9;9:1355. doi: 10.34133/research.1355 (PMC13346659; doi:10.34133/research.1355)
Supplement: Supplementary 1 — Figs. S1 to S18 Table S1 [file research.1355.f1.docx]

SUPPLEMENTARY MATERIALS

Cell wall engineered wood enabling metal-and-adhesive-free cross-laminated timber

**Fig.S1** FTIR spectra of NBW

**Fig.S2** FTIR spectra of WSM

**Fig.S3** XPS spectra of NBW and WSM


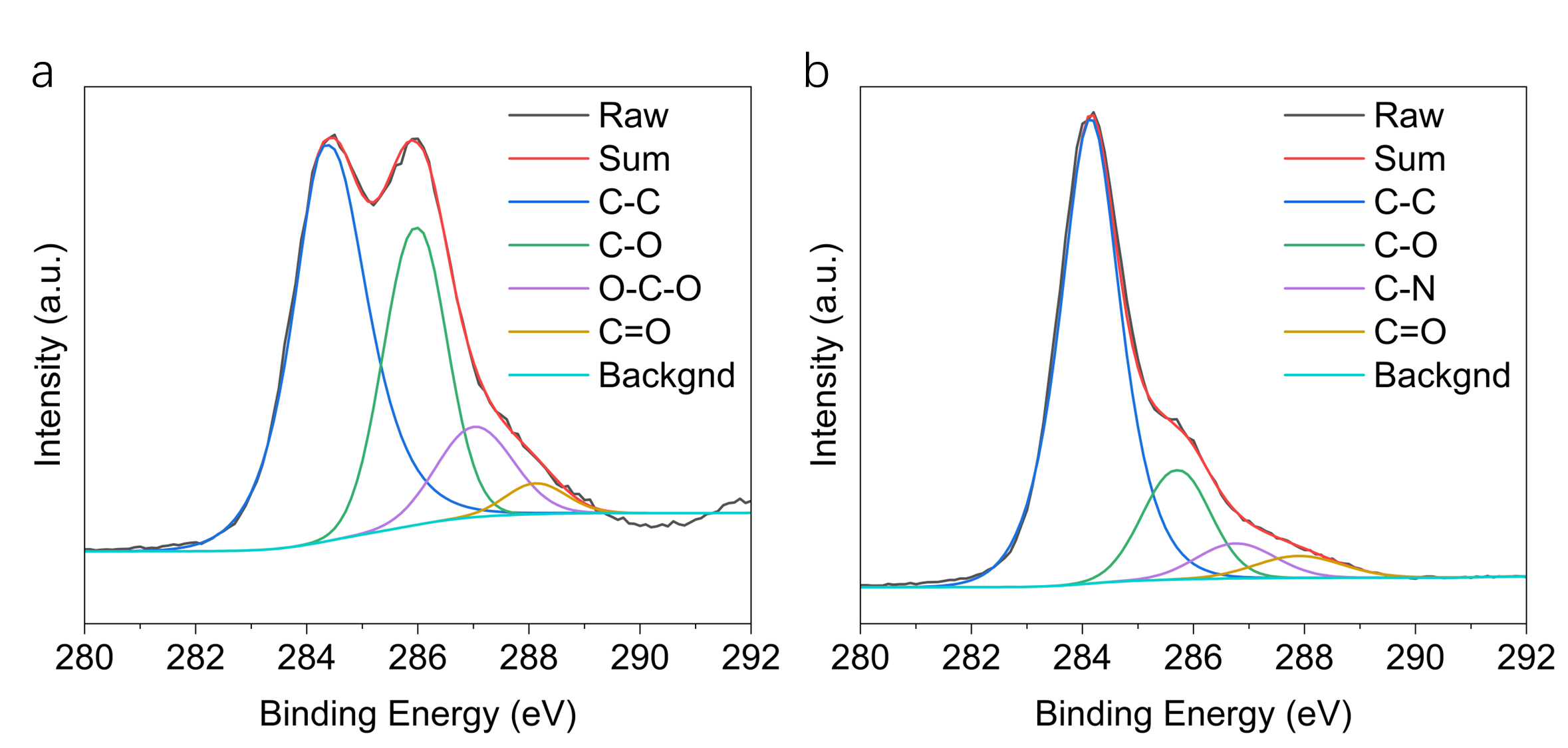


**Fig.S4** High-resolution C 1s spectra of NBW and WSM. (a) High-resolution C 1s spectra of NBW. (b) High-resolution C 1s spectra of WSM.


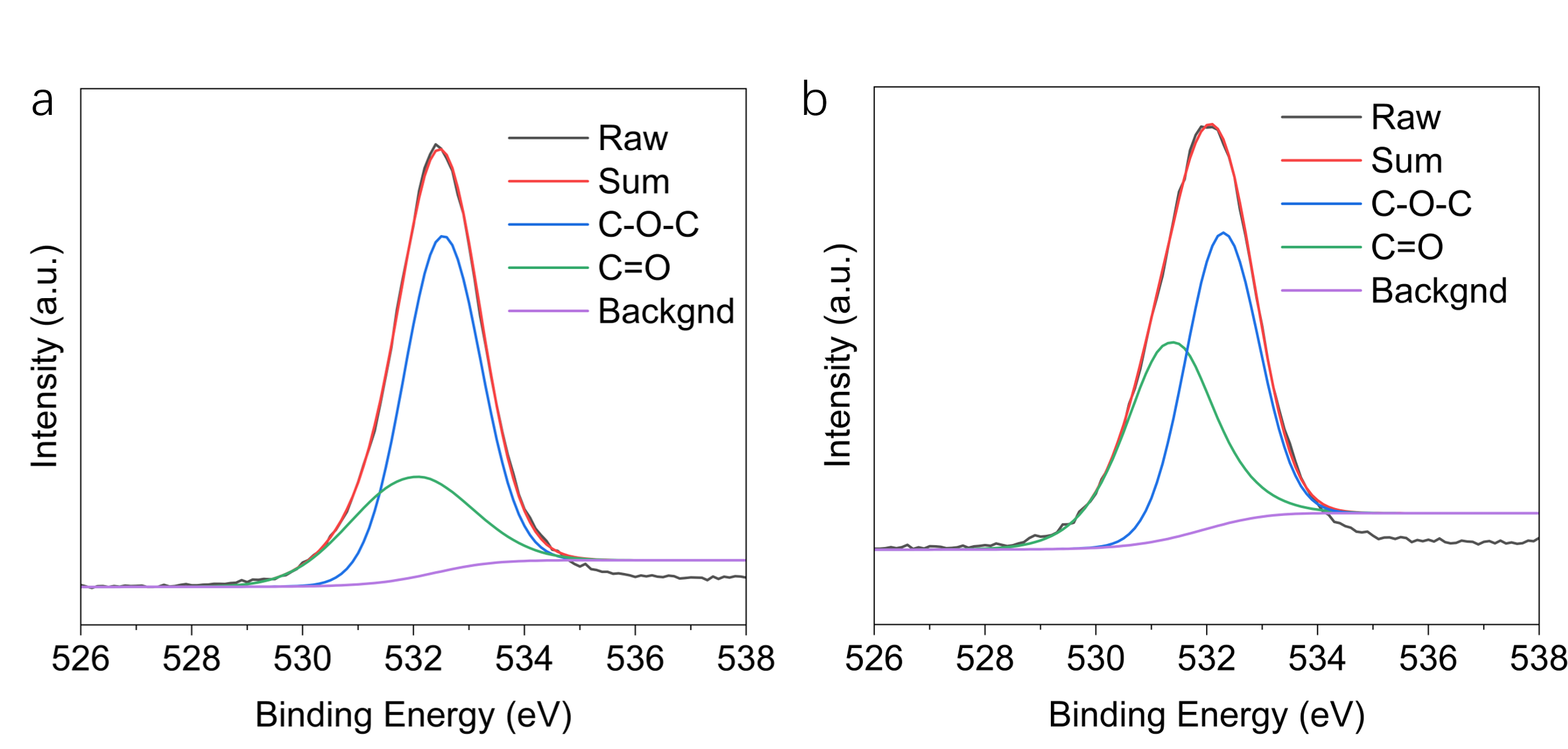


**Fig.S5** High-resolution O 1s spectra of NBW and WSM. (a) High-resolution O 1s spectra of NBW. (b) High-resolution O 1s spectra of WSM.

Tab.S1 Atomic % OF NBW and WSM

| **Peak Table** | | |
| --- | --- | --- |
| **Sample** | **NBW** | **WSM** |
| **Atomic %** | 71.74 | 81.35 |
|  | 28.26 | 18.65 |

**Fig.S6** Flexural strain-stress curves of NBM and WSM

**Fig.S7** Axial compressive strain-stress curves of NBM and WSM

**Fig.S8** Janka hardness force-displacement curves of NBM and WSM (Experimental snapshot of Janka hardness of the NBM and WSM)

**Fig.S9** Flexural force-displacement curves of wooden nail.


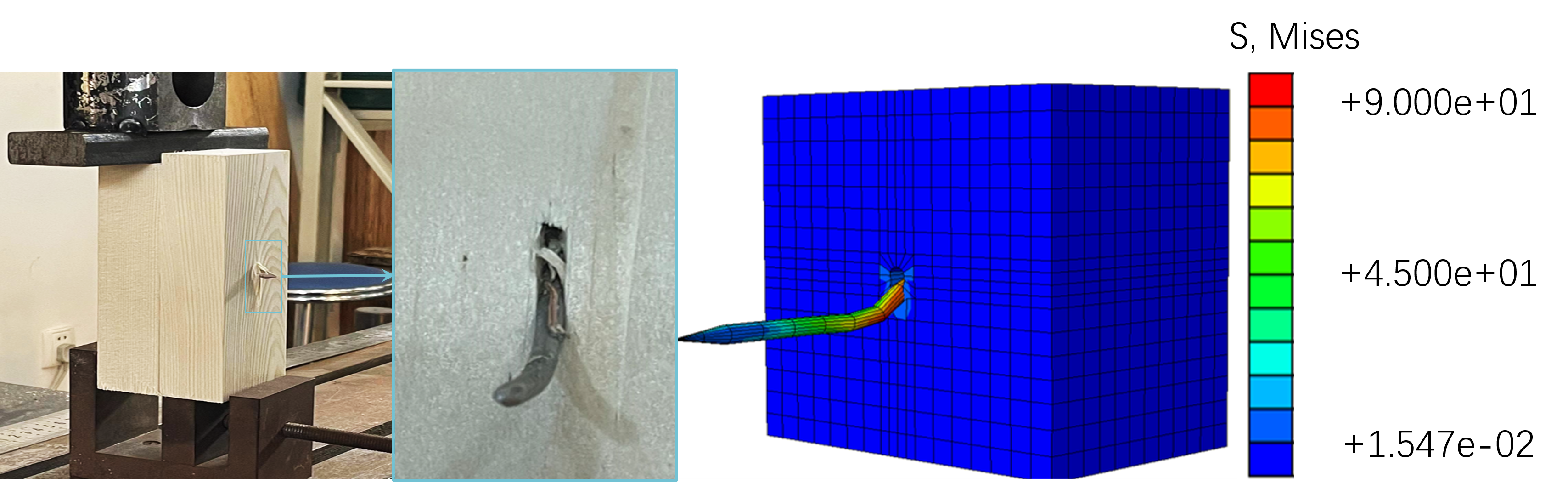


**Fig.S10** Shear strength test of steel nail-cross-laminated timber (NCLT) and finite element simulation results.

**
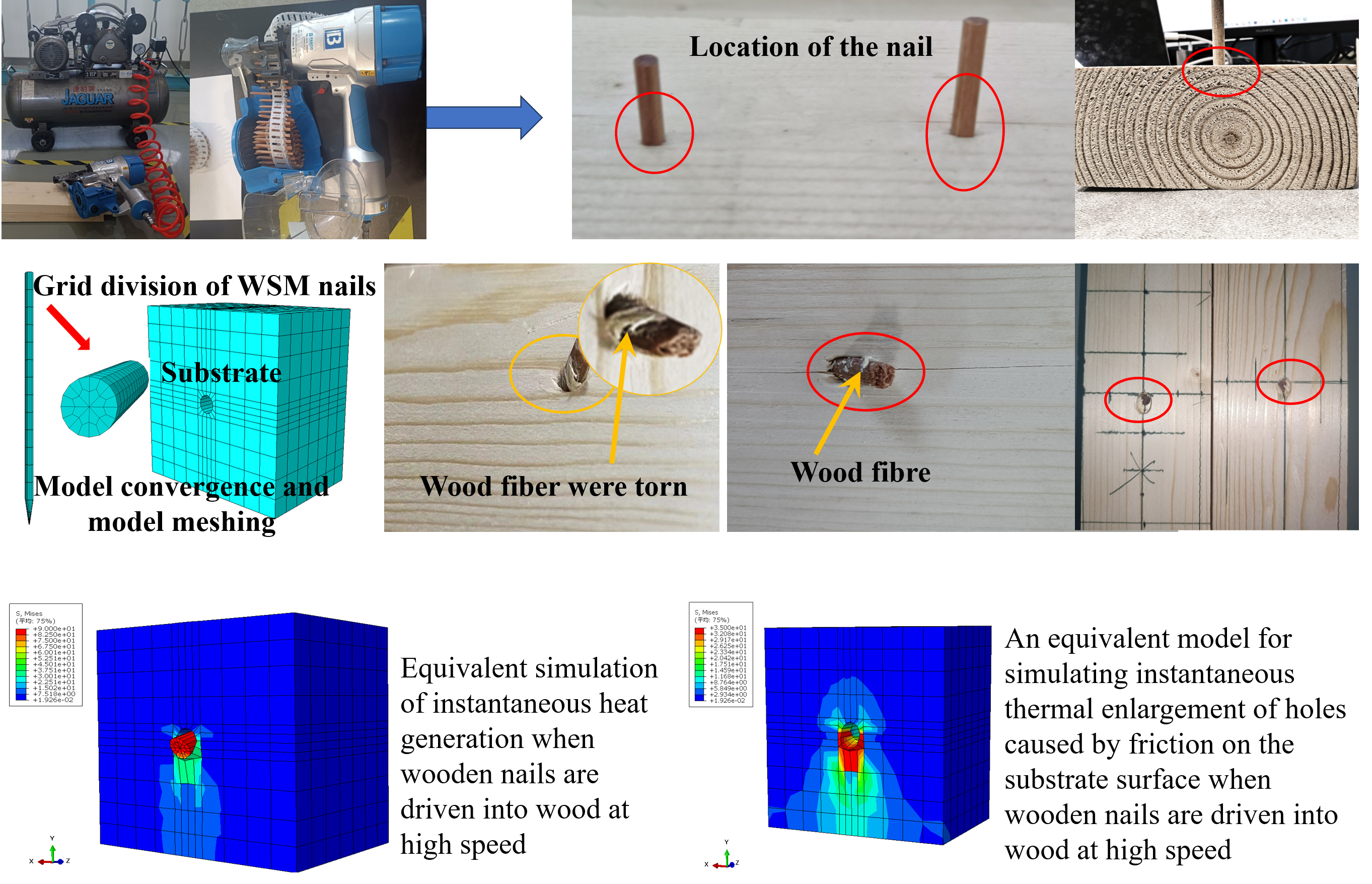
**

**Fig.S11** Finite element analysis following removal testing, model convergence, and model meshing, along with residual fibres on the surface of the WSM nails after removal tests.


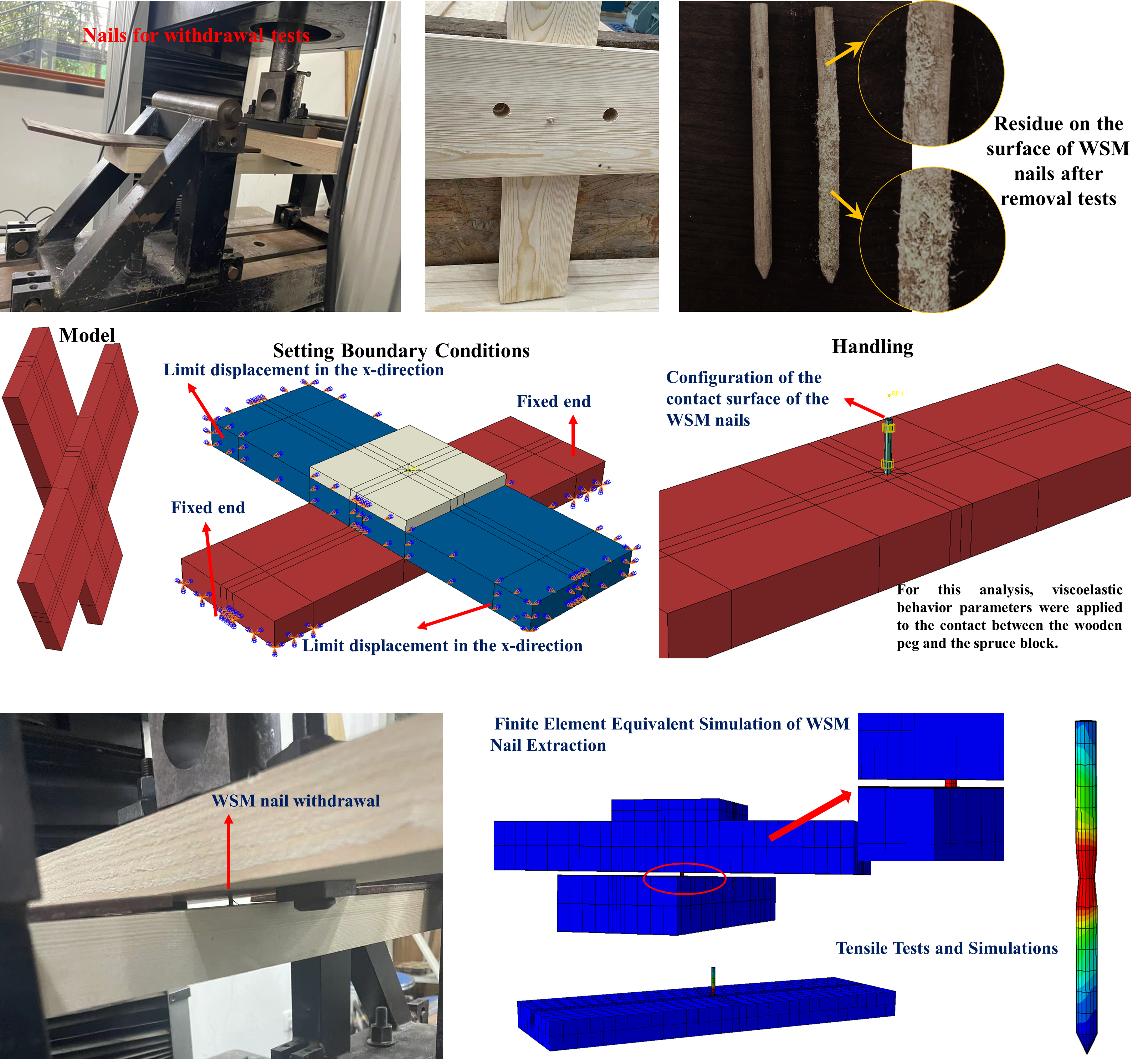


**Fig.S12 Comparison of Tensile Tests and Simulations**

**Fig.S13** TG curves of NBW and WSM.

**Fig.S14** DTG curves of NBW and WSM.

**Fig.S15** DSC curves of NBW and WSM.

**Fig.S16** DMA curves of NBW and WSM.

**Fig.S17** Densities of ^1^NBW, ^2^ MUF-treated poplar wood, ^3^ MUF-treated douglas fir, ^4^ Alkali-treated NBW, and ^5^ MUF-treated NBW

**Fig.S18** Flexural strength and bending moment of ^1^NBW, ^2^ MUF-treated poplar wood, ^3^ MUF-treated douglas fir, ^4^ Alkali-treated NBW, and ^5^ MUF-treated NBW.
